# Comparative efficacy of rTMS on different targets in Alzheimer’s disease: a systematic review and meta-analysis

**DOI:** 10.3389/fnagi.2025.1536573

**Published:** 2025-04-22

**Authors:** Yushu Zhang, Ke Dong, Jiajia Yang, Qifan Guo, Yan Zhao, Xiaoxia Zhu, Dongxu Liu, Peng Liu

**Affiliations:** ^1^Department of Rehabilitation Medicine, The First Affiliated Hospital, Sun Yat-sen University, Guangzhou, China; ^2^Guangdong Provincial Clinical Research Center for Rehabilitation Medicine, Sun Yat-sen University, Guangzhou, China

**Keywords:** repetitive transcranial magnetic stimulation, Alzheimer’s disease, cognitive function, stimulation targets, meta-analysis

## Abstract

**Background:**

Repetitive transcranial magnetic stimulation (rTMS) is emerging as a promising non-invasive intervention for Alzheimer’s disease (AD), yet therapeutic outcomes remain inconsistent across studies. This meta-analysis aimed to evaluate the cognitive benefits of rTMS in AD patients, with a specific focus on stimulation targets and protocols variations.

**Methods:**

A systematic literature search was conducted in PubMed, Web of Science, Embase, and Cochrane Library for relevant English-language studies published up to 31 May 2024. Cognitive outcomes were assessed using the Mini-Mental State Examination (MMSE) and Alzheimer’s Disease Assessment Scale-Cognitive Section (ADAS-Cog). Data were pooled using a random-effects model, with standardized mean difference (SMD) or mean differences (MD) and 95% confidence intervals (CI) calculated. Subgroup analyses were performed to examine the effects of stimulation targets, protocol variations and population demographics on rTMS efficacy.

**Results:**

Twenty-two studies involving 874 participants were included in this meta-analysis. Overall, rTMS significantly improved cognitive function (SMD = 0.27; 95% CI = 0.14–0.41; *p* < 0.0001), showing that the efficacy of rTMS varied by stimulation target and protocol. Stimulation of the dorsolateral prefrontal cortex (DLPFC) led to significant cognitive improvement (SMD = 0.49, 95% CI = −0.26 to 0.73; *p* < 0.0001), whereas bilateral DLPFC stimulation showed no significant improvement (SMD = 0.13; 95% CI = −0.40 to 0.66; *p* = 0.62). Stimulating the parietal lobe or associated regions produced moderate cognitive benefits (SMD = 0.29; 95% CI = 0.03–0.55; *p* = 0.03). Notably, multi-target stimulation over the bilateral DLPFC, parietal lobes, Wernicke’s area, and Broca’s area also showed substantial cognitive improvement (MD = 2.85; 95% CI = 1.69–4.00; *p* < 0.00001). Additionally, subgroup analysis based on geographical background revealed greater effects in studies conducted in Asia (SMD = 0.40, 95% CI = 0.14–0.65; *p* < 0.003).

**Conclusion:**

rTMS is an effective intervention for cognitive enhancement in AD, with its efficacy significantly influenced by stimulation target and protocol. Notably, the greater cognitive benefits observed in Asian populations suggest a potential role of genetic and demographic factors that warrant further investigation. These findings contribute to the development of optimized, personalized rTMS protocols for AD treatment.

**Systematic review registration:**

https://www.crd.york.ac.uk/PROSPERO/recorddashboard, CRD42023434084.

## Introduction

Alzheimer’s disease (AD) is the most common cause of dementia (Plassman et al., 2007), emerging as one of the most pressing global health challenges of the 21st century. Currently, approximately 50 million people worldwide are affected by AD, with projections indicting a rise to 150 million by 2050 (Lane et al., 2018). AD is characterized by a progressive decline of cognitive functions (e.g., memory, language, executive function, and visuospatial skill) that are accompanied by widespread disruptions in functional connectivity within crucial neural networks (Chou et al., 2022). Despite decades of research, pharmacological interventions remain largely ineffective in mitigating disease progression, offering only symptomatic relief with minimal impact on underlying neurodegenerative mechanisms (Canter et al., 2016; Livingston et al., 2017). This therapeutic gap highlights the urgent need to explore and develop alternative, more effective treatment strategies.

A growing body of research suggests that AD is not only a disorder of neuronal loss but also a disease of large-scale network dysfunction, affecting both structural and functional connectivity (Dennis and Thompson, 2014). Specifically, impaired connectivity within major neural networks, including the default mode network (DMN), dorsal attention network (DAN), salience network (SAL), executive control network (ECN), and sensory-motor network (SMN), has been strongly linked to cognitive deterioration in AD (Brem et al., 2020). The DMN, which includes the posterior cingulate cortex, precuneus, medial prefrontal cortex, inferior parietal lobule, and bilateral temporal cortex, has received particular attention due to its important role in memory consolidation, self-referential thinking, and cognitive processing (Horn et al., 2014; Whitfield-Gabrieli and Ford, 2012). Studies have revealed a close relationship between functional connectivity abnormalities in the DMN and cognitive impairments in AD. For example, Talwar et al. (2021) found that AD patients exhibited widespread impairment of functional connectivity, with the precuneus and posterior cingulate cortex being severely affected. Similarly, Zheng et al. (2019) reported disruptions in the posterior parts of the DMN in AD patients. In addition, Sorg et al. (2007) found that patients with mild cognitive impairment (MCI) exhibited impaired connectivity in the left posterior cingulate cortex and the right medial prefrontal cortex of the DMN as well as the bilateral superior parietal lobules and inferior frontal gyri of the ECN. Interestingly, compensatory increase in ECN connectivity was observed in AD patients (Agosta et al., 2012). These studies highlight the complex interplay between cognitive impairments in AD and disruptions in network connectivity. Therefore, modulating these disrupted neural networks holds potential for improving cognitive function in AD patients (Pennisi et al., 2011).

Non-invasive brain stimulation (NIBS) techniques have emerged as promising tools for modulating cortical excitability (Di Pino et al., 2014) and inducing neuroplasticity (Cirillo et al., 2017). Among these techniques, repetitive transcranial magnetic stimulation (rTMS) has received significant attention for its ability to modulate neural activity and functional connectivity (Brignani et al., 2008; Maeda et al., 2000), offering a therapeutic potential for neurological and psychiatric disorders (Esposito et al., 2022). The neurophysiological effects of rTMS are highly dependent on stimulation parameters, including frequency, intensity, target, and duration. High-frequency rTMS (HF-rTMS, ≥ 10 Hz) and intermittent theta-burst stimulation (iTBS) are known to induce long-term potentiation (LTP)-like plasticity, enhancing synaptic strength. In contrast, low-frequency rTMS (LF-rTMS, ≤ 1 Hz) and continuous theta-burst stimulation (cTBS) typically induce long-term depression (LTD)-like plasticity, reducing cortical excitability (Fox et al., 1997). Note that conventional rTMS faces limitations in spatial focality, as the widely used international 10–20 EEG-based targeting approach contributes to variability in treatment outcomes. Neuronavigation-guided rTMS is thus increasingly employed, integrating individualized neuroimaging data to optimize stimulation precision and maximize therapeutic outcomes (Lefaucheur et al., 2014; Lefaucheur et al., 2020; Terao and Ugawa, 2002).

A growing body of evidence supports rTMS as a promising intervention for neurodegenerative disorders including AD (Anderkova and Rektorova, 2014; Hsu et al., 2015; Luber and Lisanby, 2014; Zhang W. et al., 2022), showing sustained cognitive improvements in memory, attention, and executive function that can persist beyond the treatment phase (Sabbagh et al., 2020). To date, six meta-analyses have examined the cognitive effects of rTMS in AD patients (Dong et al., 2018; Liao et al., 2015; Lin et al., 2019; Wang et al., 2020; Wei Z. et al., 2022; Zhang T. et al., 2022). While these studies generally support the therapeutic effects of HF-rTMS in enhancing cognitive function, their findings exhibited considerable variability due to heterogeneity in stimulation parameters such as frequency, intensity, target, and duration. Notably, much attention has been given to optimizing rTMS frequencies, but fewer studies have systematically examined the role of stimulation target. Single-site rTMS over the DLPFC has shown efficacy in improving memory and executive functions in AD patients (Wang et al., 2014). However, emerging evidence suggests that alternative targets, such as the parietal lobe, may also play a crucial role in modulating cortical-hippocampal networks essential for memory function (Wang et al., 2014). Despite these promising findings, there remains a lack of a systematic comparisons evaluating the cognitive benefits of rTMS across different cortical regions in AD. To address this gap, we conducted a comprehensive systematic meta-analysis to assess the cognitive effects of rTMS over different cortical targets in AD patients. By examining how different stimulation targets influence cognitive improvements, this study provides critical insights for refining rTMS protocols, thereby guiding the development of optimized, personalized neuromodulation strategies for effective treatment of cognitive impairment in AD.

## Materials and methods

### Search strategy

This study protocol was registered in the PROSPERO database (CRD42023434084) and conducted in accordance with the Preferred Reporting Items for Systematic Reviews and Meta-Analyses (PRISMA) guidelines, specifically following the Cochrane extension statement. A systematic literature search was performed across PubMed, Web of Science, and EMBASE databases to identify relevant studies published between 1 January 2010 and 31 May 2024. The search strategy incorporated controlled vocabulary and free-text keywords using the following query structure: (Alzheimer’s disease OR Alzheimer Dementia OR related MeSH entry terms) AND (Transcranial Magnetic Stimulation OR Magnetic Stimulation, Transcranial OR related MeSH entry terms) AND (filters for maximum sensitivity in identifying controlled trials).

### Inclusion and exclusion criteria

Studies were included in this meta-analysis if they met the following criteria: (1) participants were diagnosed with AD based on clinical diagnostic criteria; (2) rTMS was the sole intervention; (3) cognitive function was assessed as the primary outcome measure; (4) study design included either a parallel-group or crossover sham-controlled group; (5) published in English in a peer-reviewed journal; (6) patients were allowed to continue their standard medication regimens, provided these remained unchanged throughout the rTMS treatment. Initial screening was conducted based on titles and abstracts, and full-text review was performed for studies with unclear eligibility. Studies were excluded if they were irrelevant, lacked a sham-control condition, or did not report cognitive outcomes. If relevance or eligibility remained uncertain, the full text of the paper was reviewed. Additionally, conference abstracts, case reports, and non-peer-reviewed publications were systematically excluded.

### Primary outcomes

The primary outcome measure was cognitive function, assessed using the Mini-Mental State Examination (MMSE) and the Alzheimer’s Disease Assessment Scale-Cognitive Subscale (ADAS-Cog). The analysis focused on evaluating the effect of rTMS on cognitive function across different stimulation targets. For continuous outcome data, results were synthesized using the mean and standard deviation (Mean ± SD) of changes in cognitive measurements following rTMS administration.

### Evaluation of risk of bias

The risk of bias in the included studies was assessed using a modified Cochrane Risk of Bias tool. The assessment focused on the following factors: (1) adherence to standardized diagnostic criteria for AD; (2) random sequence generation and allocation concealment; (3) blinding of participants and study personnel; (4) blinding of outcome assessments; (5) baseline comparability between rTMS and sham groups; and (6) completeness of data reporting, including dropout rates and handling of missing data. Each study was assigned a qualitative risk rating for each domain: Low risk (1), High risk (0), or Unclear (unreported information). Studies with higher cumulative scores were considered to have a lower overall risk of bias. In cases of discrepancies in risk assessment, a third independent reviewer (YJJ) was consulted to reach a consensus.

### Data analysis

All statistical analyses were performed using RevMan 5.3 software (Review Manager of Cochrane Collaboration) and R language. For continuous outcomes, effect sizes were expressed as either the standardized mean difference (SMD) or mean difference (MD) with 95% confidence intervals (CIs). Heterogeneity among studies was assessed using the I^2^ statistic, where I^2^ < 50% indicated low heterogeneity and a fixed-effects model was applied. I^2^ > 50% indicated substantial heterogeneity, resulting in the use of a random-effects model unless heterogeneity could be reduced through subgroup or sensitivity analyses. If heterogeneity persisted despite these adjustments, results were reported descriptively. Forest plots were used to visually represent the findings, with each study displayed as a colored circle (red, pink or blue) indicating SMD, MD, or risk ratio, while the overall pooled effect size was shown as a hollow orange diamond.

## Results

### Search and selection of studies

**FIGURE 1:**
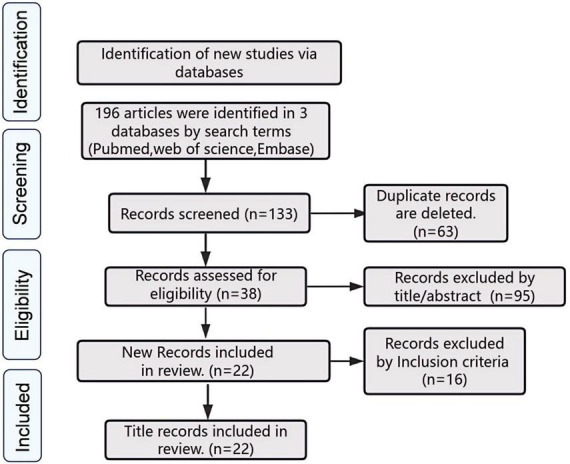
Flow diagram showing the search and selection procedure that was used for this meta-analysis.

A comprehensive literature research across PubMed, Web of Science, EMBASE, and the Cochrane Library yielded 196 studies. After removing duplicates and screening titles and abstracts for relevance, 38 articles were selected for full-text review. Following rigorous eligibility assessment, 22 studies (Ahmed et al., 2012; Bagattini et al., 2020; Brem et al., 2020; Chen et al., 2023; Cotelli et al., 2011; Hu et al., 2022; Jia et al., 2021; Koch et al., 2018; Lee et al., 2016; Leocani et al., 2020; Li et al., 2021; Padala et al., 2020; Rabey et al., 2013; Saitoh et al., 2022; Vecchio et al., 2022; Wei L. et al., 2022; Wu et al., 2015; Wu et al., 2022; Yao et al., 2022; Zhang et al., 2019; Zhao et al., 2017; Zhou et al., 2022) met the inclusion criteria and were included in the meta-analysis. The study selection process is detailed in Figure 1, and the primary clinical and demographic characteristics of the included studies are summarized in Table 1.

**TABLE 1:** Overview of studies included in the meta-analysis.

References	Country	No. of participants		Mean age (y)		Treatment duration (wk)	Intervention method		Target	Outcome indicator
		**Experimental**	**Sham**	**Experimental**	**Sham**		**Experimental**	**Sham**		
Ahmed et al., 2012	Egypt	15	15	65.9 ± 5.9	68.3 ± 4.9	1 week	20 Hz-rTMS	Sham	Bilateral DLPFC	MMSE
Ahmed et al., 2012	Egypt	15	15	68.6 ± 6.7	68.3 ± 4.9	1 week	1 Hz-rTMS	Sham	Bilateral DLPFC	MMSE
Youichi Saitoh-120%, 2022 (Saitoh et al., 2022)	Japan	15	12	76.2	75.8	4 weeks	10 Hz-120% RMT-rTMS	Sham	Bilateral DLPFC	MMSE, ADAS-Cog
Youichi Saitoh-90%, 2022, (Saitoh et al., 2022)	Japan	13	12	77.2	75.8	4 weeks	10 Hz-90% RMT-rTMS	Sham	Bilateral DLPFC	MMSE, ADAS-Cog
Bagattini et al., 2020	Italy	27	23	73.56 ± 4.91	73.35 ± 1.09	4 weeks	20 Hz-rTMS + COG	Sham + COG	Left DLPFC	MMSE
Cotelli et al., 2011	Italy	5	5	71.2 ± 6.1	74.4 ± 3.8	2 weeks	20 Hz-rTMS	Sham	Left DLPFC	MMSE
Li et al., 2021	China	37	38	65.97 ± 8.47	64.58 ± 7.88	6 weeks	20 Hz-rTMS	Sham	Left DLPFC	MMSE, ADAS-Cog
Padala et al., 2020	United States	9	11	74.3 ± 5.7	79.6 ± 7.7	4 weeks	iTBS-rTMS	Sham	Left DLPFC	MMSE
Wu et al., 2022	China	24	23	66.46 ± 8.25	66.35 ± 7.99	2 weeks	iTBS-rTMS	Sham	Left DLPFC	MMSE
Yao et al., 2022	China	15	12	63.87 ± 6.85	67.60 ± 7.88	4 weeks	5 Hz-rTMS	Sham	Bilateral cerebellum	ADAS-Cog
Koch et al., 2018	Italy	7	7	70.0 ± 5.1	70.0 ± 5.1	2 weeks	20 Hz-rTMS	Sham	Bilateral PC	MMSE
Lee et al., 2016	Korea	18	8	72.1 ± 7.6	70.3 ± 4.8	6 weeks	10 Hz-rTMS + COG	Sham + COG	Six-location	MMSE, ADAS-Cog
Zhao et al., 2017	China	17	13	69.3 ± 5.8	71.4 ± 5.2	6 weeks	20 Hz-rTMS	Sham	Bilateral parietal and posterior temporal	MMSE, ADAS-Cog
Hu et al., 2022	China	21	21	76.86 ± 6.07	75.33 ± 5.73	4 weeks	40 Hz-rTMS	Sham	Bilateral AG	MMSE, ADAS-Cog
Chen et al., 2023	China	18	6	66.67 ± 7.48	67.17 ± 8.75	4 weeks	20 Hz-rTMS	Sham	Left AG	MMSE
Jia et al., 2021	China	35	34	71.41 ± 8.85	73.41 ± 7.73	2 weeks	10 Hz-rTMS	Sham	Left parietal	MMSE
Wei L. et al., 2022	China	29	27	70.00 ± 8.63	71.67 ± 7.16	2 weeks	10 Hz-rTMS	Sham	Left lateral parietal	MMSE
Zhang et al., 2019	China	15	13	69.00 ± 8.19	68.54 ± 7.93	4 weeks	10 Hz-rTMS + COG	Sham + COG	Left DLPFC and lateral parietal	MMSE
Leocani et al., 2020	Italy	16	12	69.7 ± 7.9	72.6 ± 8.3	4 weeks	10 Hz-rTMS with H-coil	Sham	Bilateral frontal-parietal-temporal lobes	ADAS-Cog
Wu et al., 2015	China	26	26	71.4 ± 4.9	71.9 ± 4.8	4 weeks	20 Hz-rTMS	Sham	Left DLPFC	ADAS-Cog
Rabey et al., 2013	Israel	7	8	72.6 ± 8.9	75.4 ± 9.07	6 weeks	10 Hz-rTMS + COG	Sham + COG	Six-location	ADAS-Cog
Brem et al., 2020	Switzerland	16	10	69.25 ± 6.80	69.10 ± 5.24	6 weeks	10 Hz-rTMS + COG	Sham + COG	Six-location	ADAS-Cog
Vecchio et al., 2022	Italy	30	17	71.07 ± 1.25	72.24 ± 2.29	6 weeks	10 Hz-rTMS + COG	Sham + COG	Six-location	ADAS-Cog
Zhou et al., 2022	China	33	32	70 (64, 76)	74 (63.25, 79)	4 weeks	20/1 Hz-rTMS	Sham	Bilateral DLPFC	ADAS-Cog

DLPFC, dorsolateral prefrontal cortex; AG, angular gyrus; PC, precuneus; six-location includeright/left dorsolateral prefrontal cortex, right/left parietal lobe or its associated areas, Wernicke area and Broca’s area; MMSE, Mini-Mental State Examination; ADAS-Cog, Alzerimer’s Disease Assessment Scale - Cognative Section; COG, cognative training.

### Global cognitive function (immediately after the intervention)

**FIGURE 2:**
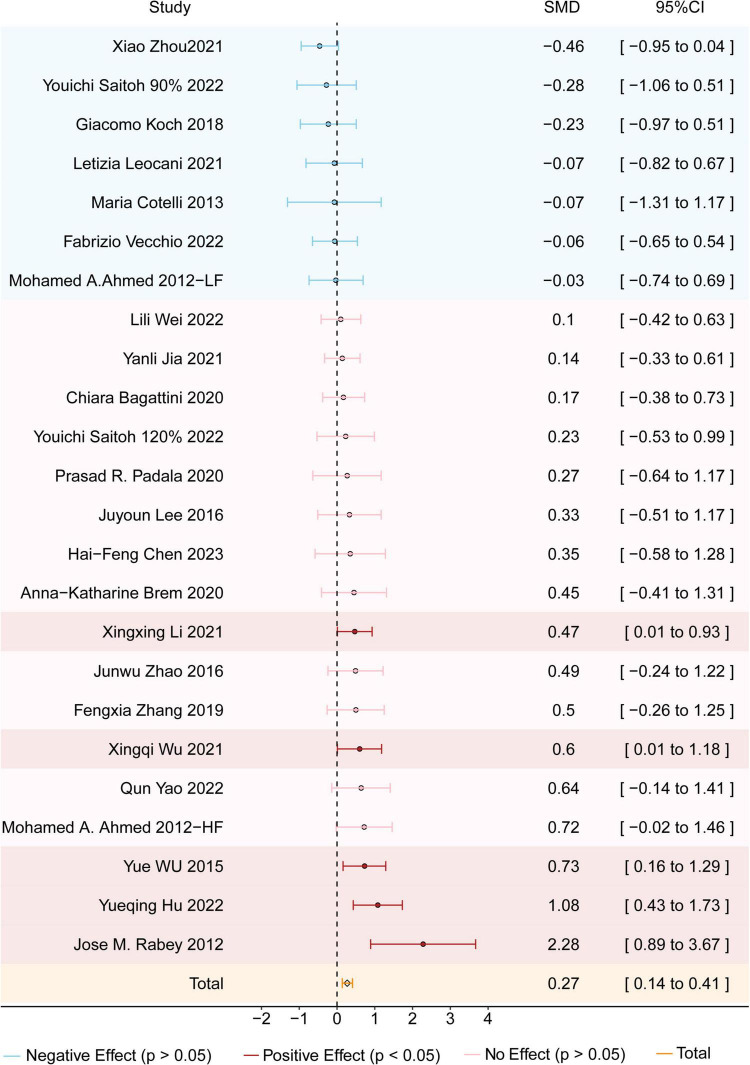
Forest plot shows the SMD (Standardized Mean Difference) and 95% CI (Confidence Interval) for all included studies compared to the sham group. Different effects are color-coded for clarity: red indicates a positive effect, blue signifies a negative effect, and pink denotes no effect. The orange diamond symbolizes the overall effect. If the diamond does not intersect the line of no effect, the overall effect is statistically significant; otherwise, it is not statistically significant.

A total of 22 studies encompassing 874 participants with AD evaluated the effects of rTMS on global cognitive function. If both MMSE and ADAS-Cog were reported, MMSE was prioritized as the primary outcome measure. The results showed that rTMS significantly improved cognitive function score in AD patients, with an SMD of 0.27 (95% CI = 0.14–0.41; z = 3.95; *p* < 0.0001) and moderate heterogeneity (I^2^ = 38%; *p* < 0.03) (see Figure 2). To further explore the potential sources of heterogeneity, additional subgroup analyses were conducted considering stimulation targets, rTMS protocols, and genetic backgrounds.

### Subgroup analysis of global cognitive function (rTMS on different targets)

**FIGURE 3:**
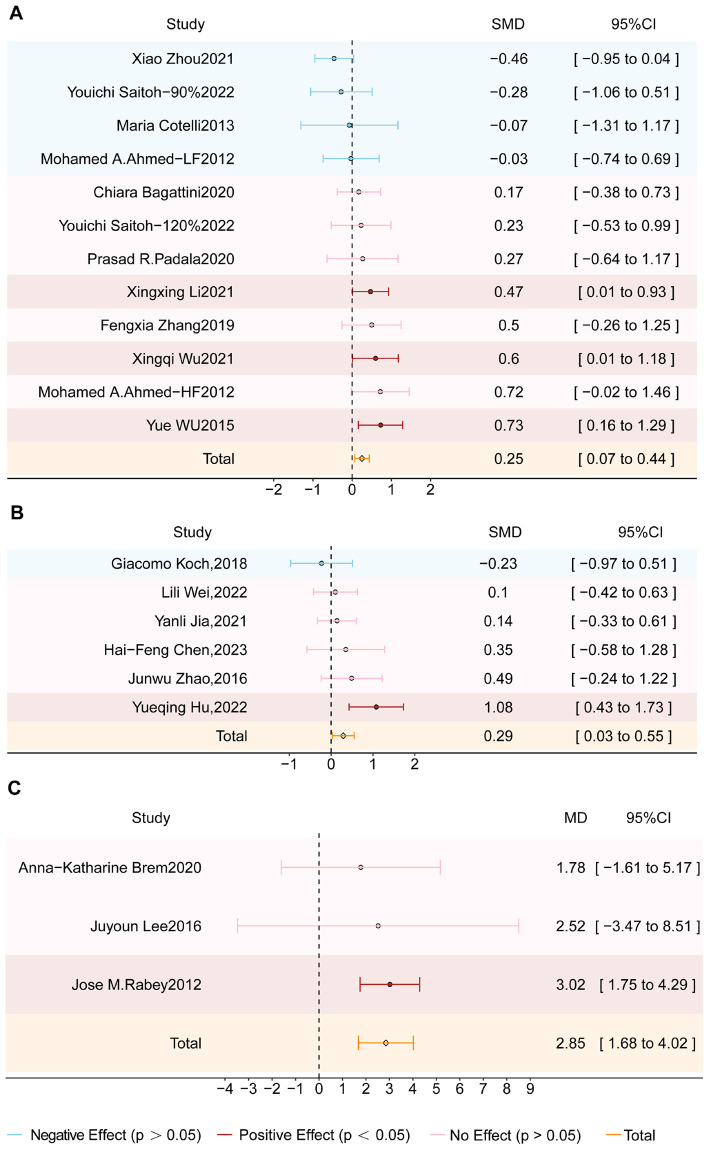
Forest plot shows the SMD (Standardized Mean Difference) or the MD (Mean Difference) and 95% CI (Confidence Interval) for three subgroups: **(A)** Dorsolateral prefrontal cortex (DLPFC), **(B)** Parietal lobe or associated regions, and **(C)** Multi-target, each compared to the sham group. Different effects are color-coded for clarity: red indicates a positive effect, blue signifies a negative effect, and pink denotes no effect. The orange diamond symbolizes the overall effect. If the diamond does not intersect the line of no effect, the overall effect is statistically significant; otherwise, it is not statistically significant.

Subgroup analyses were conducted to assess the effects of rTMS over different stimulation targets. Across all three subgroups, the experimental group showed significantly greater improvement in global cognitive function than the sham group (*p* < 0.05) (see Figure 3). DLPFC stimulation resulted in an SMD of 0.25 (95% CI = 0.07–0.44; z = 2.67; *p* = 0.008) with moderate heterogeneity (I^2^ = 37%; *p* = 0.09). Stimulation over the parietal lobe or its associated areas showed a slightly greater effect, with an SMD of 0.29 (95% CI = 0.03–0.55; z = 2.22; *p* = 0.03) and moderate heterogeneity (I^2^ = 42%; *p* = 0.12). Additionally, multi-target stimulation over the bilateral DLPFC, Broca’s area, Wernicke’s area, and bilateral parietal lobes yielded the greatest cognitive improvement, with an MD of 2.85 (95% CI = 1.69–4.02; z = 4.78; *p* < 0.00001) and low heterogeneity (I^2^ = 0%; *p* = 0.79).

### Subgroup analysis of global cognitive function (rTMS on different protocols)

**FIGURE 4:**
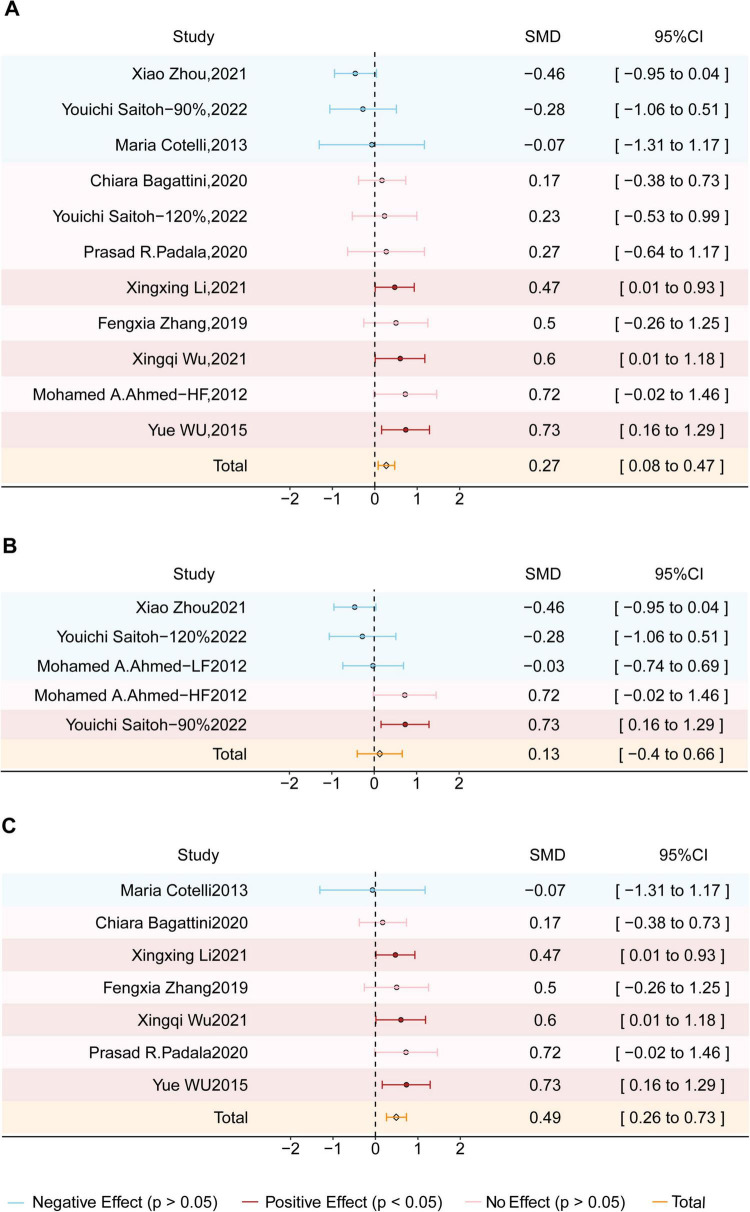
Forest plot shows the SMD (Standardized Mean Difference) and 95% CI (Confidence Interval) for three subgroups: **(A)** Excitatory stimulation, **(B)** Bilateral dorsolateral prefrontal cortex (DLPFC) stimulation, and **(C)** Left DLPFC stimulation, each compared to the sham group. Different effects are color-coded for clarity: red indicates a positive effect, blue signifies a negative effect, and pink denotes no effect. The orange diamond symbolizes the overall effect. If the diamond does not intersect the line of no effect, the overall effect is statistically significant; otherwise, it is not statistically significant.

The effects of different stimulation protocols were further analyzed (see Figure 4). Excitatory rTMS significantly improved cognitive function, with an SMD of 0.27 (95% CI = 0.08–0.47; z = 0.78; *p* = 0.005) and moderate heterogeneity (I^2^ = 41%; *p* = 0.08). Bilateral DLPFC stimulation did not show improvement compared to the sham group, with an SMD of 0.13 (95% CI = −0.40–0.66; z = 0.49; *p* = 0.62) and high heterogeneity (I^2^ = 70%; *p* = 0.01). In contrast, left DLPFC stimulation yielded significant cognitive improvement, with an SMD of 0.49 (95% CI = 0.26–0.73; z = 4.11; *p* < 0.0001) and low heterogeneity (I^2^ = 0%; *p* = 0.78).

### Subgroup analysis of global cognitive function (rTMS on different genetic backgrounds)

**FIGURE 5:**
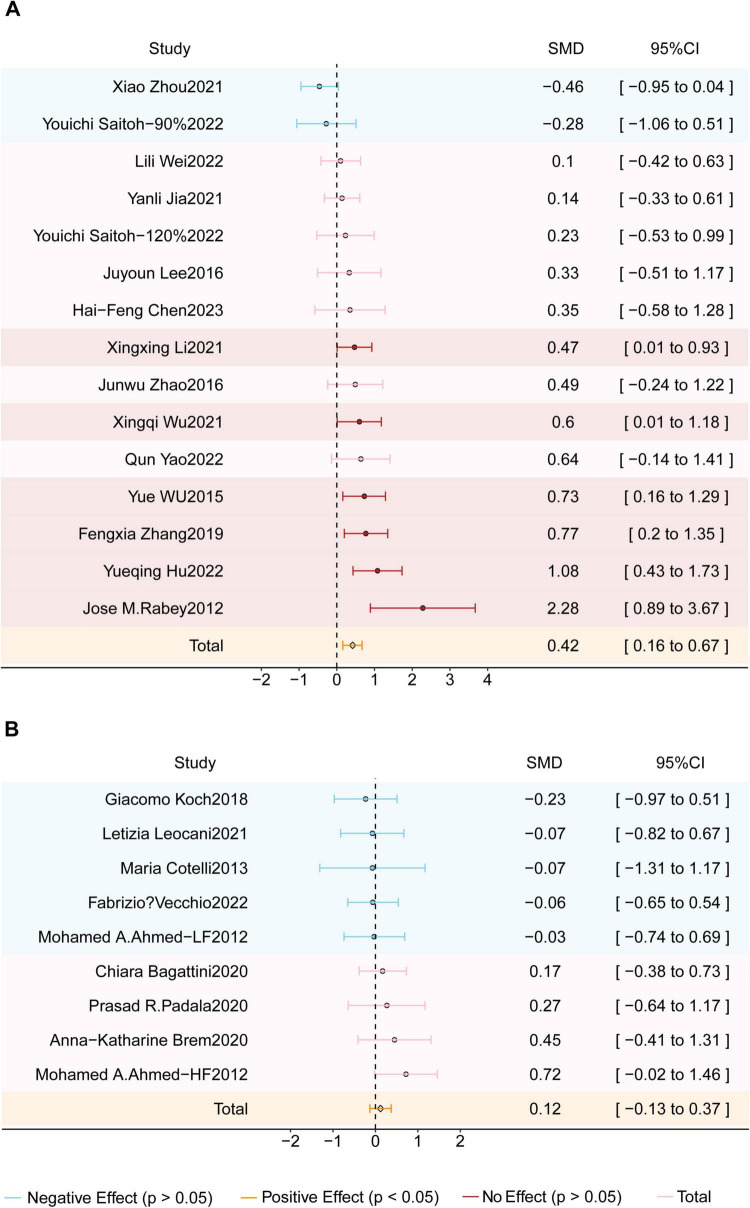
Forest plot shows the SMD (Standardized Mean Difference) and 95% CI (Confidence Interval) for two subgroups: **(A)** the Asian studies group and **(B)** the Europe-America-Africa studies group, each compared to the sham group. Different effects are color-coded for clarity: red indicates a positive effect, blue signifies a negative effect, and pink denotes no effect. The orange diamond symbolizes the overall effect. If the diamond does not intersect the line of no effect, the overall effect is statistically significant; otherwise, it is not statistically significant.

To evaluate the impact of genetic background, subgroup analyses were conducted based on geographic origin (see Figure 5). Among the included studies, 14 were conducted in Asia, one in Africa, six in Europe, and one in North America. The Asian subgroup demonstrated a significant cognitive benefit, with an SMD of 0.42 (95% CI = 0.16–0.67; z = 3.21; *p* = 0.001) and moderate heterogeneity (I2 = 56%; *P* = 0.004). In contrast, studies conducted in Europe, Africa, and North America did not yield significant effects, which may be due to variability in sample size, treatment protocols, and genetic factors.

### Risk of bias assessment

**FIGURE 6:**
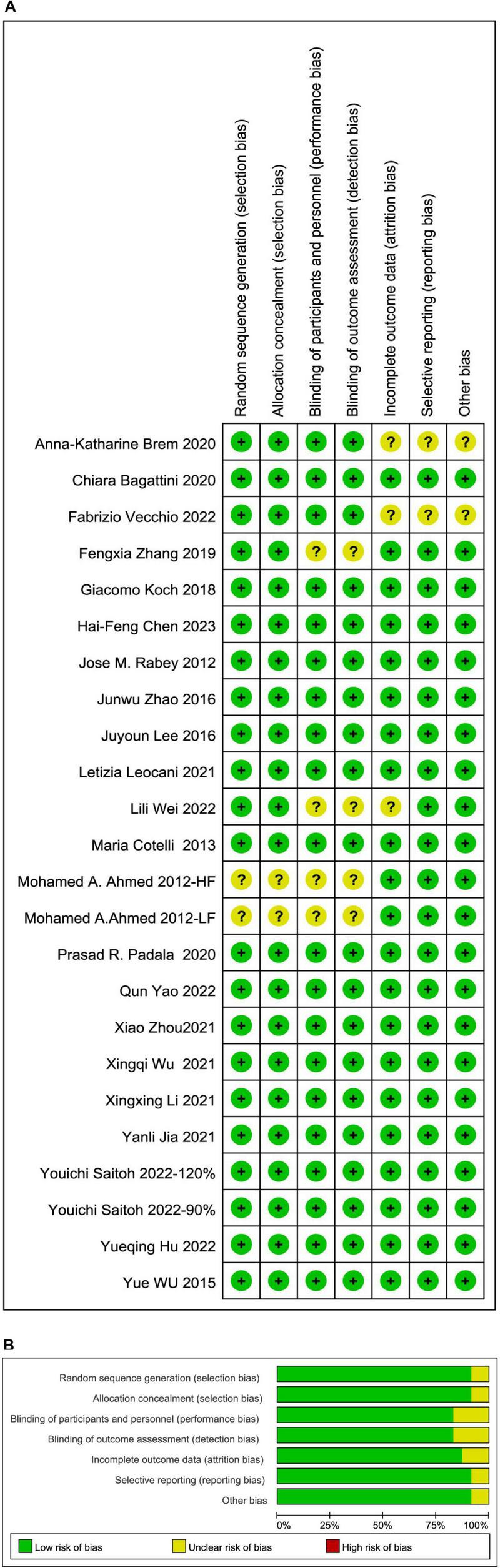
**(A)** Risk of bias summary and **(B)** Risk of bias graph present the quality of included studies.

The risk of bias assessment was conducted independently by two reviewers, following the Cochrane Intervention Systematic Review Manual 5.1.0. Any discrepancies were resolved through consultation with a third reviewer. Overall, the included studies were rated as having low to moderate risk of bias, as summarized in Figure 6.

## Discussion

This systematic meta-analysis, synthesizing data from 22 randomized, sham-controlled trials involving 874 participants, provided a comprehensive evaluation of the efficacy of rTMS on cognitive function in AD. Our findings confirmed that rTMS significantly enhanced global cognitive performance, with therapeutic effects varying based on stimulation targets. Notably, unilateral DLPFC and parietal lobe stimulation produced moderate cognitive improvements, whereas bilateral DLPFC stimulation did not yield significant effects. In contrast, multi-target rTMS over the bilateral DLPFC, parietal lobes, Wernicke’s area, and Broca’s area produced the most pronounced cognitive benefits. These findings demonstrate the potential of rTMS as a therapeutic intervention for AD, with multi-site stimulation strategies offering greater efficacy than single-site approaches by enhancing network-level plasticity and promoting functional connectivity across cognitive domains.

AD is characterized by profound cognitive impairments, accompanied by widespread structural and functional disruptions in brain networks. Alterations in functional connectivity between the parietal and frontal cortices have been closely linked to cognitive decline in AD (Jia et al., 2021; Velioglu et al., 2021). The parietal lobe, particularly the precuneus, plays an important role in the cortical-hippocampal network that supports memory processing and higher-order cognitive functions (Cavanna and Trimble, 2006). Likewise, the DLPFC is a key hub in the DMN and the ECN essential for working memory, executive function, and cognitive control (Andrews-Hanna et al., 2010; Cai et al., 2017; Cavanna and Trimble, 2006; Crippa et al., 2011). Given the progressive dysfunctions of these networks observed in AD, rTMS over these key regions offers a promising approach to enhancing functional connectivity and attenuating cognitive decline.

Our meta-analysis showed that HF-rTMS over the left DLPFC was effective in improving cognitive function in AD patients. However, several studies (Ahmed et al., 2012; Saitoh et al., 2022; Zhou et al., 2022) employing bilateral DLPFC stimulation yielded inconsistent results, potentially due to differences in stimulation parameters. For example, Ahmed et al. (2012) examined the effects of 20, 1 Hz, or sham rTMS over the bilateral DLPFC, showing that HF-rTMS significantly improved cognitive function whereas LF-rTMS did not elicit cognitive enhancement. These findings suggest that excitatory rTMS is more effective in AD, possibly due to its role in promoting LTP-like plasticity and enhancing synaptic efficiency. Similarly, Saitoh et al. (2022) reported that HF-rTMS over the bilateral DLPFC resulted in cognitive improvements, while another study (Cotelli et al., 2006) found enhanced action naming abilities following bilateral DLPFC stimulation. The neural basis for these effects may involve rTMS-induced modulation of functional connectivity across the DMN, ECN, and FPN, thereby strengthening network integration and cognitive performance (Yuan et al., 2021).

Notably, differential effects of unilateral DLPFC stimulation have also been reported. Zhou et al. (2022) found that 20 Hz rTMS over the left DLPFC combined with 1 Hz rTMS over the right DLPFC led to significant cognitive improvements. This finding aligns with research suggesting that inhibitory rTMS over the right DLPFC may counteract dysfunctional network activity, thereby restoring functional balance in patients with MCI (Fregni and Pascual-Leone, 2007). Given that the right DLPFC is involved in cognitive inhibition (Anderson et al., 2004), LF-rTMS over this region has been shown to reduce hyperactive frontoparietal activity, suppress DMN overactivation (Cui et al., 2019; Wang et al., 2012), and ultimately enhance episodic memory (Turriziani et al., 2019) and recognition performance (Turriziani et al., 2012).

The present study incorporated six studies (Chen et al., 2023; Hu et al., 2022; Jia et al., 2021; Koch et al., 2018; Wei L. et al., 2022; Zhao et al., 2017) that examined the effects of rTMS over the parietal regions such as the left parietal cortex, bilateral parietal lobes, bilateral angular gyrus (AG), and precuneus. Neuroimaging evidence highlights the critical role of the parietal cortex in cognitive processing, particularly in memory consolidation, language processing, and large-scale network integration. Notably, HF-rTMS over the AG has been shown to significantly enhance functional connectivity between the AG and language-related regions such as the left inferior frontal gyrus (IFG) and anterior middle temporal gyrus (aMTG) (Garcia et al., 2022). This network-level modulation may underlie the rTMS-induced improvements in verbal fluency and semantic processing observed in AD patients. Additionally, HF-rTMS over the left AG has been found to increase connectivity with the dorsal medial prefrontal cortex (dMPFC), which plays a fundamental role in memory retrieval and executive function (Chen et al., 2023). There is evidence showing that parietal lobe stimulation induces multiple neurophysiological changes, including increased regional cerebral blood flow, enhanced cortical-subcortical connectivity, and activation of residual hippocampal neurons, all of which contribute to improved synaptic plasticity (Wang et al., 2014). These effects further support the therapeutic potential of this novel stimulation target (Jia et al., 2021). Beyond the AG, the precuneus has emerged as another promising stimulation site due to its central role in episodic memory retrieval (Nellessen et al., 2015). Structural and functional neuroimaging studies have consistently shown that AD patients exhibit reduced precuneus thickness, abnormal task-related activation, and impaired functional connectivity within this region (Chen et al., 2017). Notably, HF-rTMS over the precuneus has been shown to restore DMN connectivity (Wei L. et al., 2022) and modulate long-term memory function (Bonnì et al., 2015; Koch et al., 2018), providing a potential basis for cognitive improvement in AD patients.

Our findings support and extend previous meta-analytic evidence demonstrating the beneficial effects of rTMS on cognitive functions in AD. A large-scale meta-analysis incorporating nine clinical trials with 361 participants demonstrated that rTMS over the DLPFC significantly improved cognitive function in AD patients (Zhang T. et al., 2022). Two additional studies provide compelling evidence that rTMS over the left lateral parietal cortex enhanced functional connectivity within neural networks and improved memory-related cognitive outcomes (Jia et al., 2021; Velioglu et al., 2021). These converging findings underscore the therapeutic potential of combining DLPFC and parietal cortex stimulation, suggesting that multi-target rTMS protocols may be more effective than single-site stimulation for cognitive restoration in AD.

Notably, one study (Yao et al., 2022) included in our meta-analysis explored the effects of HF-rTMS over the bilateral cerebellum, showing that rTMS-induced neuroplastic changes in cerebellar nodes selectively enhanced connectivity with key cortical regions, particularly the DLPFC, cingulate cortex, and medial frontal cortex. These network-level enhancements were associated with significant cognitive improvements, particularly in memory consolidation and language processing. These findings highlight the role of the cerebellum role in higher-order cognitive functions, suggesting that its extensive connectivity with cerebral cortical networks may contribute to rTMS-induced cognitive benefits in AD.

Cognitive impairment in AD is predominantly driven by deficits in synaptic plasticity and neural network dysfunction within the DMN (Brier et al., 2012; Grieder et al., 2018; Menon, 2023). This network encompasses key regions such as the frontal lobes (affected by cholinergic neuron degeneration), the cingulate cortex, posterior parietal areas (including the precuneus), and temporal regions (Boublay et al., 2016; Mohan et al., 2016). Applying rTMS to these regions may enhance network connectivity and functional integration, thereby supporting cognitive improvements in AD patients. Neuroimaging studies provide further insights into the network-level alterations observed in AD. For example, AD-related network disruptions primarily involve bilateral prefrontal-parietal disconnections, as well as disrupted dominant-hemisphere connectivity between posteroinferior frontal and superior temporal regions (Alcalá-Lozano and Garza-Villarreal, 2018; Nguyen et al., 2018).

Our meta-analysis provides one of the first comprehensive evaluations of multi-target rTMS protocols in AD treatment. The subgroup analysis included three studies employing multi-target stimulation, specifically targeting the bilateral DLPFC, Broca’s and Wernicke’s areas, and bilateral parietal somatosensory association cortex (pSAC). These studies followed a structured sequential stimulation approach, selecting three distinct targets daily with no repetition of stimulation sites across consecutive days. Participants underwent 30 treatment sessions over 6 weeks (five sessions/week). While Brem et al. (2020), applied suprathreshold stimulation to all six targets, other studies (Lee et al., 2016; Rabey et al., 2013) employed subthreshold stimulation for Broca’s area and bilateral DLPFC, while using suprathreshold stimulation for other targets. These findings demonstrate significant cognitive improvements across multiple domains, including syntax processing, grammatical comprehension, and spatial memory. The neurobiological mechanisms underlying multi-target rTMS-induced cognitive improvements may involve several neural pathways. The frontal lobe is integral to episodic memory processing (Budson and Price, 2005), while the medial temporal lobe regulates recent memory formation. Additionally, the left prefrontal cortex is critical for verbal working memory tasks (Barbey et al., 2013). Accumulating evidence suggests that rTMS can enhance synaptic plasticity through repeated stimulation of specific neural circuits, leading to cognitive improvements (Brem et al., 2020; Casarotto et al., 2023; Luber and Lisanby, 2014) and strengthening functional connectivity (Johansen et al., 2014). Further, enhanced functional connectivity between the orbitofrontal cortex, DLPFC, and parietal regions facilitates the integration of rewards processing, executive control, and spatial attention during reinforcement learning (Jarbo and Verstynen, 2015).

A large-scale meta-analysis of 831 fMRI studies focused on DLPFC activation patterns demonstrated significant co-activation between the left DLPFC and several key regions, including the right DLPFC, bilateral pSAC, and the anterior cingulate cortex (Alcalá-Lozano and Garza-Villarreal, 2018). These findings highlight the potential of rTMS applied to specific cortical sites to modulate functionally connected neural networks, thereby enhancing cognitive and linguistic capabilities. This effect is thought to be mediated by mechanisms of LTP and LTD, both of which are fundamental to synaptic plasticity and network reorganization (Andersen et al., 2017). HF-rTMS applied in a multi-target sequential stimulation paradigm has been shown to increase synaptic efficacy, elicit LTP-like effects, and strengthen interregional connectivity (Buch et al., 2011; Koch et al., 2013). These mechanisms suggest that network-based rTMS approaches may drive lasting cognitive improvements in AD by enhancing synaptic plasticity and restoring disrupted functional connectivity.

## Limitations

This study has several limitations that should be acknowledged. First, the limited number of included studies and small sample sizes constrained the scope of our subgroup analyses, particularly regarding the differential effects of rTMS on distinct cognitive domains. Second, we did not distinguish between left- and right-hemispheric stimulation or different frequency protocols (e.g., HF- vs. LF-rTMS), factors that may contribute to variability in treatment efficacy. Third, cognitive outcomes were predominantly assessed using the MMSE and ADAS-Cog. These measures exhibit variations in sensitivity and reliability across different stages of AD, which may lead to an overestimation or underestimation of the therapeutic effects of rTMS. Future studies incorporating multimodal cognitive assessments, functional neuroimaging markers, and standardized rTMS protocols are essential for refining our understanding of optimal stimulation targets and individual treatment responses in AD.

## Conclusion

This meta-analysis provides a comprehensive synthesis of existing evidence on the effects of rTMS on cognitive function in AD. Despite variability across studies, our findings demonstrate that rTMS targeting the DLPFC and parietal lobe leads to significant cognitive improvements. Notably, multi-target rTMS stimulation engaging both prefrontal and parietal regions enhances cognitive outcomes more effectively than single-site stimulation. These results underscore the therapeutic promise of targeted rTMS interventions in mitigating AD-related cognitive decline. Future research should focus on optimizing stimulation protocols, integrating neuroimaging-guided targeting, and exploring individualized approaches to maximize the clinical efficacy of rTMS in the treatment of AD.

## Data Availability

The raw data supporting the conclusions of this article will be made available by the authors, without undue reservation.
